# Lentiviral vector-mediated transduction of adult neural stem/progenitor cells isolated from the temporal tissues of epileptic patients

**DOI:** 10.22038/IJBMS.2019.42285.9983

**Published:** 2020-03

**Authors:** Sara Abdolahi, Azizollah Khodakaram-Tafti, Hadi Aligholi, Saeid Ziaei, Maryam Khaleghi Ghadiri, Walter Stummer, Ali Gorji

**Affiliations:** 1 Department of Pathobiology, School of Veterinary Medicine, Shiraz University, Shiraz, Iran; 2 Shefa Neuroscience Research Center, Khatam Alanbia Hospital, Tehran, Iran; 3 Department of Neuroscience, School of Advanced Medical Sciences and Technologies, Shiraz University of Medical Sciences, Shiraz, Iran; 4 Department of Basic Sciences, Faculty of Paramedical Sciences, Shahid Beheshti University of Medical Sciences, Tehran, Iran; 5 Department of Neurosurgery, Westfälische Wilhelms-Universität, Münster, Germany; 6 Epilepsy Research Center, Department of Neurology and Institute for Translational Neurology, Westfälische Wilhelms-Universität Münster, Münster, Germany; 7 Neuroscience Research Center, Mashhad University of Medical Sciences, Mashhad, Iran; 8 Department of Neuroscience, Faculty of Medicine, Mashhad University of Medical Sciences, Mashhad, Iran

**Keywords:** GFP, Lentivirus, Neural stem/progenitor – cells, Seizure, Transplantation

## Abstract

**Objective(s):**

Neural stem/progenitor cells (NS/PCs) hold a great potential for delivery of therapeutic agents into the injured regions of the brain. Efficient gene delivery using NS/PCs may correct a genetic defect, produce therapeutic proteins or neurotransmitters, and modulate enzyme activation. Here, we investigated the efficiency of a recombinant lentivirus vector expressing green fluorescent protein (GFP) for genetic engineering of human NS/PCs obtained during brain surgery on patients with medically intractable epilepsy.

**Materials and Methods:**

NS/PCs were isolated from human epileptic neocortical tissues. Three plasmids (pCDH, psPAX2, pMD2.G) were used to make the virus. To produce the recombinant viruses, vectors were transmitted simultaneously into HEk-293T cells. The lentiviral particles were then used to transduce human NS/PCs.

**Results:**

Our *in vitro* study revealed that lentivirus vector expressing GFP efficiently transduced about 80% of human NS/PCs. The expression of GFP was assessed as early as 3 days following exposure and remained persistent for at least 4 weeks.

**Conclusion:**

Lentiviral vectors can mediate stable, long-term expression of GFP in human NS/PCs obtained from epileptic neocortical tissues. This suggests lentiviral vectors as a potential useful tool in human NS/PCs-based gene therapy for neurological disorders, such as epilepsy.

## Introduction

In spite of adequate treatment with anticonvulsive medicaments, drug-resistant epilepsy affects millions of people worldwide; representing 40% of those with epilepsy (1). Therefore, innovative therapeutic approaches for epilepsy are highly needed. Among several novel therapeutic strategies, numerous stem cell transplantation and gene transfer approaches are being investigated (2). Several viral vectors have been used for the transfer and expression of genes in the brain, including adeno-associated virus, herpes simplex virus, and lentivirus (3). The application of viral vectors to deliver genes to the central nervous system (CNS) exhibits a strong potential for the mechanistic investigations and therapeutic approaches of various neurological disorders (4). These vectors may be used for treatment of epilepsy by transduction of endogenous cells expressing inhibitory modulators and reduce hyperexcitability, expressing neurotrophic factors, promote survival and repair ability of injured neurons, and/or expressing opsins for alterations in cell firing patterns (2). 

Neural stem/progenitor cells (NS/PCs) are a group of ectodermal cells, which can proliferate and differentiate into the main cell types of the CNS (5). NS/PCs are identified in the brain developmental stage as well as in adult CNS of mammalian species (6, 7). Human NS/PCs (hNS/PCs) can be differentiated into various cells, such as neurons, oligodendrocytes, and astrocytes can be used to investigate specific human genetic mutations associated with neurological disorders (8). hNS/PCs could also represent a unique source of cells for gene-based therapeutic approaches (9). Enormous investigations have focused on embryonic NS/PCs for therapeutic purposes. However, using these cells in therapeutic approaches faces to several challenges, including immunological, availability and ethical aspects (10, 11). Evidence indicates that adult stem cells can represent a promising potential source of autologous cells, which can differentiate into multiple lineages and secrete various proliferative and immune-modulating factors (12, 13). Isolation, proliferation, and differentiation of hNS/PCs from different human brain areas, including the cerebral cortex (14), the olfactory bulb (15), the subventricular zone (16), the hippocampus (17, 18), the subcortical white substance (19), and the amygdala (20) as well as pathological tissues, such as meningioma (21), have been reported. Given hNS/PCs capability to differentiate into various cells as well as their ability to integrate into host brain tissues, hNS/PCs have attracted attention as a promising source for therapeutic cell transplantation (22). 

 Over the past decades, gene therapy has benefited from parallel advances in viral vectors and delivery approaches as well as from improvements of stem cell technologies (23). These viral vectors could be used to investigate the function of various genes of NS/PCs during their differentiation into various cell fates as well as to produce differentiated cells for cell therapy (24, 25). Lentiviral vectors have been shown to successfully transduce dividing and non-dividing cells of the CNS, including neurons, oligodendrocytes, astrocytes, and neuronal stem cells, in different animal models (26). Furthermore, several investigations revealed that viral vectors can efficiently transduce both proliferating and differentiated human neural progenitor cells obtained from embryonic human brain tissues (27, 28). However, no investigation evaluated the use of lentiviral vectors to transduce adult hNS/PCs and obtain a sustained expression of transgenes. 

Therapeutic surgical resection of the mesial temporal lobe in patients suffering from medically intractable epilepsy provides a unique opportunity to obtain adult hNS/PCs from different brain regions, such as the hippocampus and the amygdala (20, 22). Using green fluorescent protein (GFP) as a marker to monitor gene expression and protein localization (29), the main purpose of this study was to evaluate whether lentivirus vectors could efficiently transduce and integrate into adult hNS/PCs derived from epileptic tissues *in vitro*. 

## Materials and Methods

**Figure 1:**
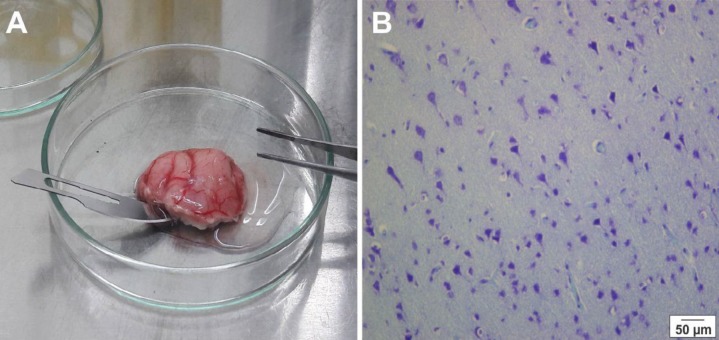
Histological verification of the temporal tissue samples. Resected temporal tissue from a patient with medically intractable epilepsy (A), and a photomicrograph of Nissl stained section of resected epileptic human cortical tissue (B)

Tissue samples were obtained during brain surgery on two patients suffering from medically intractable mesial temporal lobe epilepsy (Table 1). The brain tissues were transferred from the operating theaters to the laboratory in cold phosphate-buffered saline (PBS; Gibco, Germany) with 10% penicillin-streptomycin (pen/strep; Gibco, Germany) within 5-10 min (Figure 1). The experimental methods were approved by the Ethical Committee of the Shefa Neuroscience Research Center, Tehran, Iran. Informed consent was obtained from both patients.

***Study design***

hNS/PCs isolated from the temporal lobe specimens of patients undergone epilepsy surgery and then cultured as a sphere. Immunocytochemistry assay was performed to characterize hNS/PCs and the identity of these cells was evaluated using flow cytometry. Three plasmids were used to make the virus. To produce the recombinant viruses, the HEK-293T cells were then transfected with vectors and the transfection efficiency was evaluated by fluorescent microscopy. The lentiviral particles were then used to transduce human NS/PCs. After 72 hr as well as 4 weeks, the transduced cells were analyzed using fluorescent microscopy.

***Brain tissue dissection***

The resected mesial temporal lobe tissues with dimension of ~0.9×0.8×0.6 mm were transferred in a glass dish containing 5 ml fresh PBS and washed 2-3 times with fresh PBS to remove debris. Any visible blood vessels were gently removed from the brain tissue mass and the sample was cut into small pieces using a scalpel. Enzymatic digestion was done using accutase (Gibco, Germany) for 10 min at room temperature and the suspension was broken up by pipetting for 2-3 times. An equal volume of fresh medium was added to the tube and mixed gently to stop the enzymatic activity. Then, the suspension was centrifuged at 110 g for 5 min at room temperature. After that, the supernatant was discarded and the cells were re-suspended in 1-2 ml of Dulbecco’s modified Eagle’s medium/F12 (DMEM/F12; Gibco, Germany).

***Cell counting and plating***

Ten microliters of trypan blue 0.04% (Biomedical, USA) was added to 10 μl of the cell suspension. Next, 10 μl of the mixture was added on a hemocytometer. Cells were counted in four areas and the average cell number was calculated for each sample. The single cells were cultured in neurosphere medium DMEM/F12 containing 20 ng/ml EGF (Sigma, Germany), 20 ng/ml FGF2 (Sigma, Germany), 2 μg/ml heparin (Sigma, Germany)**, **1% L-glutamine (Sigma, Germany), 1% pen/strep (Gibco, Germany), 2% B27-Supplement (Gibco, Germany), and 1% N2-Supplement (Gibco, Germany). A suspension culture is grown in non-coated flasks at a density of 4×10^4^ cell/cm^2 ^in a humidified incubator (37 ^°^C, 5% CO_2_).

***Passaging the cells***

When the neurospheres were ready for culturing (with a diameter of 150-200 μm), the medium with suspended spheres was removed from the flasks and centrifuged at 110 g for 5 min. Cell pellet was then re-suspended in 1-2 ml (depending on the pellet size) of accutase for 10 min under the hood. An equal volume of fresh medium was added to the tube and repeatedly pipetting up and down. Then, centrifugation steps were repeated. The supernatant was removed and the neurospheres were re-suspended in an appropriate volume of medium before culturing in non-coated flasks. The numbers of spheres and cells were counted after each passage.

***Immunofluorescence assay***

To characterize isolated cells, immunocytochemistry

assay was performed against hNS/PCs markers, nestin and Sox2. The cells were grown on gelatin-covered glass coverslips, fixed by 4% paraformaldehyde (Merck, USA) in PBS for 15 min, blocked and permeabilized with 5% bovine serum albumin (Sigma, Germany) and 0.2% Triton X-100 (Sigma, Germany) in PBS for 2 hr at room temperature. The cells were incubated overnight at 4 ^°^C with mouse anti-nestin (1:50 diluted in PBS; Santa Cruz, Germany) and rabbit anti-Sox2 (1:100 diluted in PBS; Santa Cruz, Germany) primary antibody. Following three washes in PBS, the sections were incubated with goat anti-rabbit IgG (FITC) (1:1000 diluted in PBS; Abcam, UK) or goat anti-mouse IgG (FITC) (1:1000 diluted in PBS; Abcam, UK) for 1 hr at room temperature. Cell nuclei were stained with 4’, 6-diamidine-2-phenylidole dihydrochloride (DAPI; Sigma, Germany). The stained cells were photographed with an Olympus fluorescence microscope. In control experiments, the primary antibody was replaced with mouse or rabbit control IgG (Abcam, UK). 

***Flow cytometry analysis***

hNS/PCs (4×10^3^ cells/cm^2^) were cultured in 75 cm^2 ^flasks containing DMEM/F12 with supplements and 10% FBS. hNS/PCs were then harvested from 80-90% confluent monolayers using accutase for 5 min at 37 °C. Aliquots of 1×10^5^ viable cells per sample were first blocked with 3% bovine serum albumin and incubated with a panel of antibodies, including phycoerythrin (PE)-conjugated mouse anti-nestin (Santa Cruz, Germany) and PE-conjugated rabbit anti-Sox2 (Santa Cruz, Germany) at 4 °C for 30 min. For all antibodies, a relevant isotype control was used and then the cells were evaluated by BD FACSAria II (BD Biosciences, Germany). Data were analyzed by FlowJo 7.6.5 software. 

***Production of lentiviral vectors***

Three lentiviral plasmids (pCDH, psPAX2, and pMD2.G) were used to make the virus. All lentiviral vectors were handled in a class II biosafety laboratory. In the first stage, the amount of plasmid DNA increased by *Escherichia coli Stbl3*. The pCDH plasmid contained GFP, the psPAX2 plasmid carries gag and pol genes and contains proteins of the capsid, and the pMD2.G plasmid used as an envelope plasmid encoding the VSV G surface protein. The bacteria containing plasmid were grown on Luria Bertani medium for 24 hr in 37 °C in a shaker incubator in the presence of ampicillin. All three vectors were extracted from *E. coli Stbl3 *using NucleoBond Xtra Plasmid Midiprep Plasmid Extract Kit. The human embryonic kidney 293T (HEK-293T) cell line was used as a host for virus packaging. These cells were cultured in DMEM medium containing 10% FBS and 1% mixture of pen/strep antibiotics in an incubator (37 °C and 5% CO_2_). pCDH vector mixed along with the psPAX2 and pMD2.G plasmids was co-transfected into 80-90% confluent HEK-293T cells in a 6-well palate using Lipofectamine 3000 reagent (Invitrogen, USA) according to the manufacturer’s protocol. Virus particles were harvested 24 hr and 72 hr post-transfection.

***Lentiviral titration***

Approximately 6×10^4^ HEK-293T cells were seeded per well in 3-well plates and added 4, 16, and 32 µl of soup containing the virus to each cell. The medium was changed 24 hr after transduction and the GFP expression in cells were analyzed using flow cytometry at 72 hr post-transduction. The viral titration was calculated using the formula: (1×10^5^ seeded cells × %GFP positive) × 100 / µl of viral soup.

***Neural stem cell transduction***

Cells were cultured until approximately 80% confluent before passaging or transduction. Approximately 80% confluent hNS/PCs were transduced using produced virus particles at multiplicity of infection (MOI) of 51 in the presence of 10 µg/ml polybrene (Sigma, Germany). The medium was changed after 12 hr and the cells were analyzed 72 hr and 4 weeks post-transduction using a fluorescent microscope. 

## Results

***Culture of hNS/PCs***

**Figure 2:**
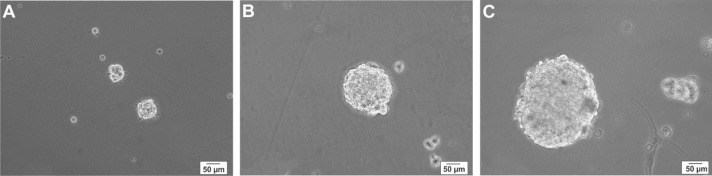
Phase-contrast images of floating neurospheres derived from human neural stem/progenitor cells in different days. Cell clusters were observed after 2 days (A), small neurospheres with the translucent center were seen after 7 days (B), and larger neurospheres with the dark center were observed after 14 days (C)

**Figure 3:**
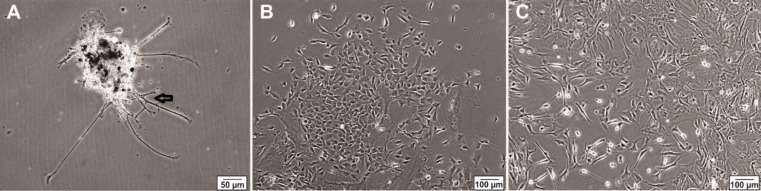
Phase-contrast images of human neural stem/progenitor cells after adhesion to coated culture flask. A few cells have started to migrate out of the sphere, inset shows a cell that have migrated from a neurosphere (arrow). (A). Phase contrast representative images of human neural stem/progenitor cells are shown in adherent conditions after passage 2 (B) and passage 3 (C)

hNS/PCs were exposed to the neurosphere medium. A few floating cell clusters were observed in suspension after 48 hr (Figure 2A). Over the next 3-5 days, the number of hNS/PCs increased. At 7-days, the center of the neurosphere was light and translucent with a diameter of 150-200 μm (Figure 2B). After two weeks, the proliferating hNS/PCs formed neurospheres that mostly measured 250–300 μm in diameter with dark center and a bright outer portion (Figure 2C). Over the course of the first two weeks, hNS/PCs preferentially expand in adherent conditions and become dense until they reach confluence. Culturing hNS/PCs in treating flasks resulted in expansion of stem cells in a monolayer manner (Figure 3).

***Characterization of hNS/PCs***

**Figure 4:**
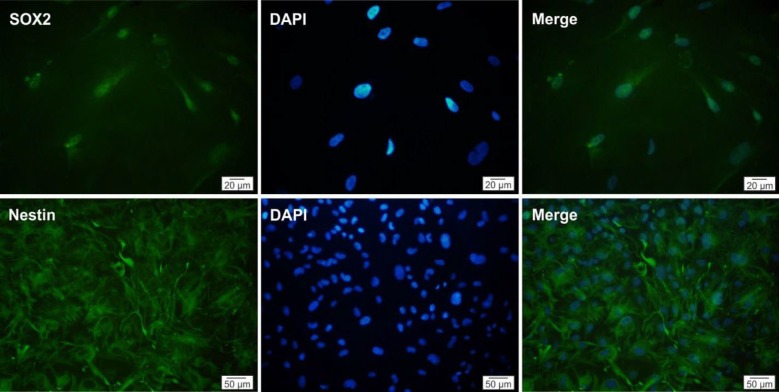
Immunocytochemical evaluation of human neural stem/progenitor cells. The cells obtained following culture of the temporal lobe specimens expressed neural stem cell markers Sox2 (green) and nestin (green). The nuclei are seen in blue color

To confirm that the cell populations that have been selected and expanded were indeed hNS/PCs, the expression of neural stem cell markers was evaluated. After the third passage, immunocytochemical characterization of hNS/PCs revealed that the majority of isolated cells expressed stem/progenitor cell markers nestin and Sox2 (Figure 4).

***Flow cytometric analysis of hNS/PCs***

**Figure 5:**
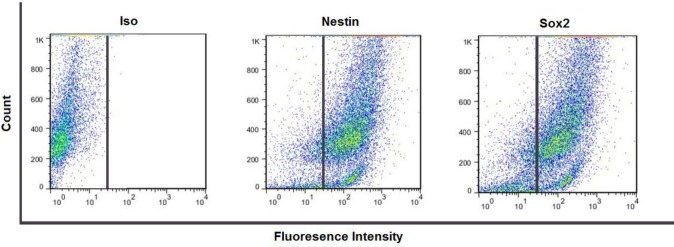
Flow cytometry analysis of nestin and Sox2 positive cell population indicates the expression of these neural stem cell markers in human neural stem/progenitor cells at passages 3 (in adherent conditions)

By the third passage of the isolated hNS/PCs culture, the identity of the cells was investigated using flow cytometry immunophenotypic analysis. hNS/PCs were stained with antibodies raised against specific antigens and their phenotypes were analyzed. Our results have shown a near-pure population of hNS/PCS expressing of 94% nestin-positive and 96% Sox2-positive cells (Figure 5).

***Production and titration of lentiviral vectors***

**Figure 6:**
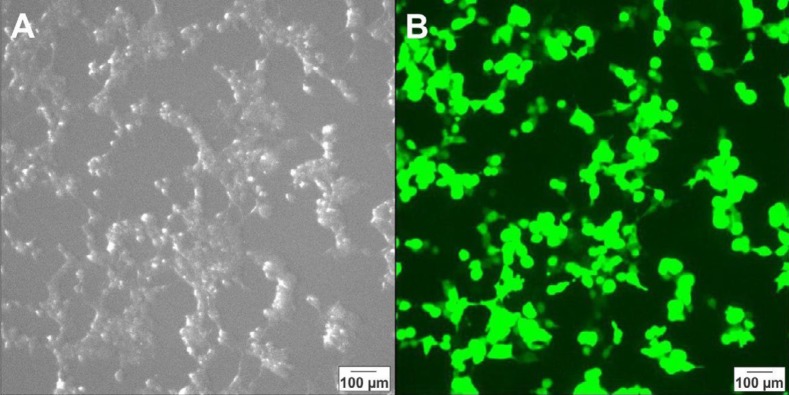
Photomicrographs of HEk-293T cells 72 hr after transfection with pCDH and helper plasmids taken by a light microscope (A) and a fluorescent microscope (B)

**Figure 7:**
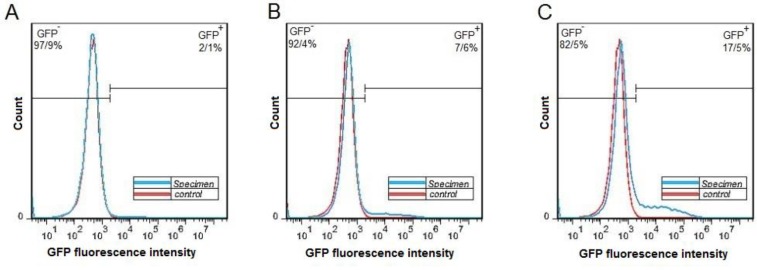
Fluorescence-activated cell sorting analysis of HEK-293T cells after transduction by lentiviruses. Histogram of the green fluorescent protein of positive and negative cells after transduction by 4 µl (A), 16 µl (B), and 32 µl of viral soup are shown (C)

For production of lentiviral particles, the HEK-293T cells were transfected by pCDH and helper vectors and the transfection efficiency was evaluated by fluorescent microscopy 24 hr after transfection (Figure 6). The cell culture medium was collected from the culture of transfected cells and the viral soup was used for lentiviral titration. The titration results showed that 2.1%, 7.6 % and 17.5% of the cells in specimen 1, 2, and 3 were GPF-positive, respectively (Figure 7). For the estimation of the virus titration, the amount of viruses in each well was measured and the mean number of viruses in three wells reported as the viral titration amount (Table 2). 

***hNS/PCs transduction***

**Figure 8:**
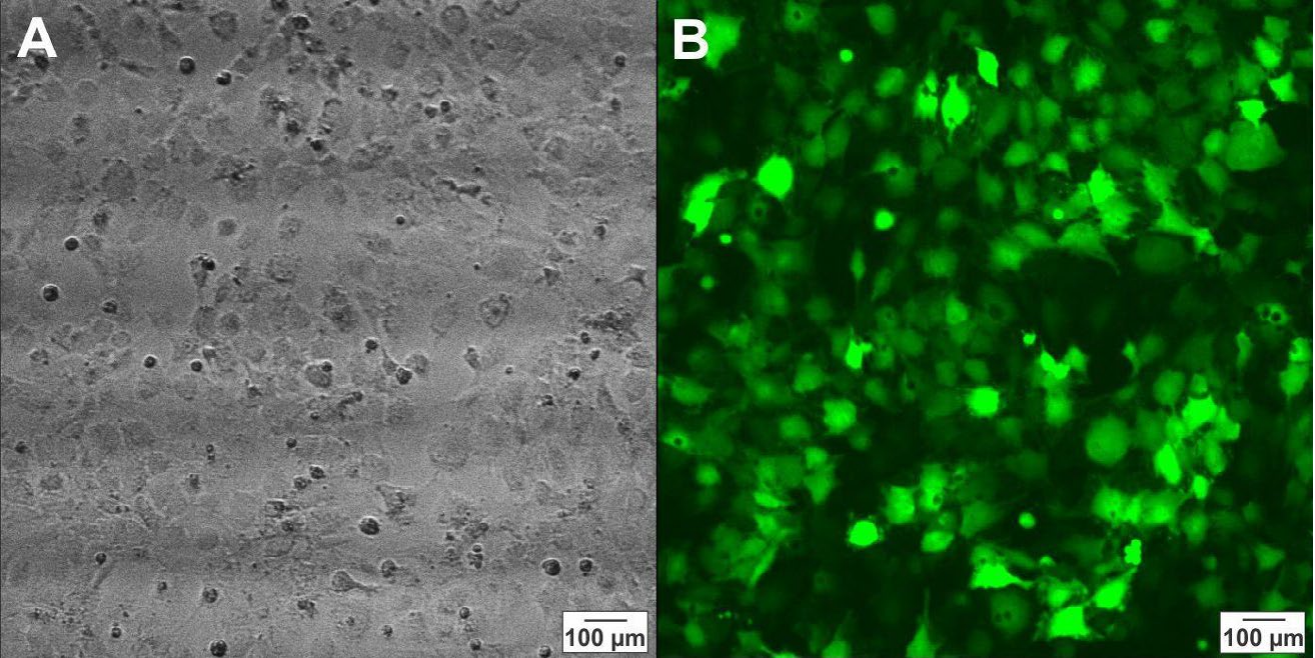
Photomicrographs of human neural stem/progenitor cells 72 hr after transduction by a virus containing green fluorescent protein taken by light microscopy (A) and fluorescent microscopy (B)

The hNS/PCs were seeded in T25 culture flask until they reached 80% confluency and 500 µl of soup containing viruses were added to the culture medium. According to the viral concentration, the cells were transduced by about MOI 51. After 72 hr, the transduced cells were analyzed by fluorescent microscopy, we counted GFP positive cells with image j software and the results revealed that about 80% of the cells were GFP-positive (Figure 8). These cells were still GFP-positive for at least four weeks after transduction.

## Discussion

In this study, we demonstrated for the first time that lentivirus vectors could efficiently and stably transduce and integrate into adult hNS/PCs derived from epileptic neocortical tissues. Transduced hNPCs were still able to survive and proliferate for a few weeks without apparent cytotoxicity *in vitro*. Our findings are in line with the previous investigations that demonstrated the ability of lentiviral vectors to transfer the luciferase-gens to human primary fetal astrocytes (24) and human fetal cortical progenitor cells as well as to several types of cells in various animal models (26, 30).

Promising evidence suggests the therapeutic potential of hNS/PCs transplantation and gene therapy in several neurological disorders, including epilepsy, amyotrophic lateral sclerosis, Alzheimer’s disease, Parkinson’s disease, and spinal cord injury (2, 31, 32). Several numbers of clinical trials have been carried out in some of these disorders, like Parkinson’s disease and Alzheimer’s disease (33, 34). In spite of numerous experimental studies, these techniques have not been assessed in clinical trials for epilepsy (35, 36). Using lentivirus, adeno-associated virus, and herpes simplex viral vectors in animal models, the most gene therapies for treatment of epilepsy targeted neurotrophic factors (37), galanin (38), adenosine kinase (39), and neuropeptide Y (40). However, the non-selective nature of these vectors is a challenging problem to direct the transgene into a specific cell type (41). Furthermore, the availability of functional and safe vectors for persistent transfer of genes to NS/PCs is essential for gene therapy of epilepsy. The present data revealed that lentiviral vectors could be efficiently targeted adult hNS/PCs obtained from drug-resistant epileptic patients. These hNS/PCs genetically modified with the efficient and safe lentivirus vectors could be an appropriate tool for mechanistic investigations and gene therapy in epilepsy. Further studies are required to explore the biosafety and efficiency of this method in *in vivo* epilepsy models.

On the other hand, for transplantation investigations, it is necessary to recognize and track the transplanted cells in recipient tissues. Adult NS/PCs are characterized by self-renewal and multi-potency, relative quiescence, and differentiation capacity, which could modulate the repair of damaged brain tissue (6). The most appropriate approach for labeling these cells depends on different criteria, including labeling efficiency, label retention, and intracellular localization (42). Our data indicated that GFP-based reporter could fulfill these criteria (43) and is an optimal marker for labeling cells for cell therapy in epilepsy. 

Gene therapy using lentiviral vectors has been currently approved for the treatment of children with acute lymphoblastic leukemia and several other cell therapy approaches using lentiviral vector are in late stage of clinical development (44). Although our experiments revealed an efficient *in vitro* lentiviral transduction of hNS/PCs, further investigations are warranted to rigorously clarify the safety of using these cells for treatment of diseases.

**Table 1:** Medical history of patients. The neocortical tissues were obtained from 2 patients with medically refractory epilepsy

**Case**	**Gender**	**Age (Year)**	**Age at the onset **	**Duration of epilepsy (Year)**	**Seizure frequency**	**AED/Drug history**	**Histology and imaging**	**Seizures**
1	Male	28	14	14	Weekly (1-2)	Valproic acid, Carbamazepine, Gabapentin	Sclerosis	GS*
2	Male	28	10	18	Weekly (2-3)	Carbamazepine, Phenytoin	Sclerosis	GS

*GS: Generalized seizures

**Table 2:** Data of recombinant lentiviral vector encoding GFP gene

**Viral soup amount**	**GFP positive cells**	**Virus titration**
4	2.1%	52*10^6^ (TU)/ml
16	7.6%	47*10^6^ (TU)/ml
32	17.5%	55*10^6^ (TU)/ml

TU: Transducing units

## Conclusion

These data demonstrated that lentivirus vectors are able to transduce hNS/PCs obtained from epileptic neocortex. These transduced hNS/PCs were still able to proliferate *in vitro*. These hNS/PCs transduced with the safe, efficient, and persistent lentivirus vectors could contribute to the investigation of basic mechanism and for diagnostic and therapeutic approaches of epilepsy.
